# Advances in Gynecological Cancers

**DOI:** 10.3390/ijms23116152

**Published:** 2022-05-31

**Authors:** Michalis Liontos, Oraianthi Fiste, Flora Zagouri, Meletios-Athanasios Dimopoulos

**Affiliations:** Department of Clinical Therapeutics, National and Kapodistrian University of Athens, Alexandra Hospital, 11528 Athens, Greece; ofiste@med.uoa.gr (O.F.); florazagouri@yahoo.co.uk (F.Z.); mdimop@med.uoa.gr (M.-A.D.)

The burden of gynecological cancer constitutes a major focus of public health efforts, as it continues to represent a significant cause of cancer mortality, exerting not only physical and emotional distress but also serious financial strain upon individuals, caregivers, and communities. Over the last decade, new insights into disease biology have led to an unprecedented development and approval of novel systemic therapeutics for ovarian, cervical, and uterine cancer. Indeed, immunotherapy and numerous targeted agents have transformed the therapeutic landscape of gynecologic malignancies, highlighting the significance of biomarker testing for personalized treatment decisions.

For ovarian cancer, first-line maintenance therapy with poly(ADP-ribose) polymerase inhibitors (PARPi), with or without the antiangiogenic monoclonal antibody bevacizumab, represents a paradigm shift. This new class of drugs prevents DNA damage repair (DDR) processes by binding to PARP-1 and subsequently leads to synthetic lethality in tumor cells that lack proficient double-stranded breaks (DSB) mechanisms, such as the homologous recombination (HR) pathway. Of note, approximately 50% of epithelial ovarian cancers exhibit HR deficiency (HRD), while BRCA1/2 mutation prevalence varies between 20 and 25%. However, primary or acquired resistance to PARPi is inevitable, with critical prognostic implications. Hence, translational studies on resistance mechanisms in these novel compounds are crucial.

Recently, immune checkpoint inhibitors (ICIs) have revolutionized the treatment of advanced, inoperable solid tumors, including several gynecologic malignancies. These molecules target programmed cell death 1 (PD-1), its ligand (PD-L1), and cytotoxic T-cell lymphocyte-associated antigen 4 (CTLA-4), activating immunomodulation. While immunotherapy has not yet demonstrated efficacy in ovarian cancer, either as monotherapy or in combinatorial approaches, it has shown durable responses in endometrial and cervical cancer, fueling hope and drastically reshaping their management. Remarkably, a variety of biomarkers, including microsatellite instability-high status (MSI-H), PD-L1 expression, tumor mutation burden (TMB), and T-cell infiltration levels, rather than tumor histotype, seem to define treatment’s efficacy. Therefore, an in-depth understanding of the tumor microenvironment has been comprehensively explored, whereas the identification of predictive biomarkers has been intensively studied.

In this first edition of the Special Issue “Advances in Gynecological Cancers”, current advances in the field of molecular landscape, the prognosis, diagnosis, and treatment of gynecologic malignancies, as well as novel therapeutic strategies and future directions are presented. Troitskaya, O. et al. [1] demonstrated that a high expression of epidermal growth factor receptor (EGFR) in the MCF7 cell line transformed the adherent cell culture into a three-dimensional (3D) in vitro cell culture, which is known to closely resemble the in vivo disease environment, providing a rather accurate model for cancer therapeutics efficacy testing. Moreover, MCF7-EGFR spheroids, which represent self-assembled cancer cell aggregates, seem to induce plasticity with regards to cellular markers, such as human epidermal growth factor receptor 3 (HER3), determining drug resistance and tumor progression. Gralewska, P. et al. [2] examined the combination therapy of PARPi olaparib with the anti-hyperglycemic biguanide metformin on HR-proficient epithelial ovarian cancer cell lines. Interestingly, increased reactive oxygen species (ROS) production could contribute to the underlying mechanism of sensitization of BRCA wild-type tumor cells to the targeted therapy by metformin. The authors concluded that such preclinical observations provide a rationale for further investigation of metformin repurposing for the treatment of ovarian cancer. Promising anticancer activity has also been provided by the ornithine decarboxylase (ODC) inhibitor, difluoromethylornithine (DFMO), whether used alone or in combination with cisplatin, in epithelial ovarian cancer cell line [3].

Metastasis-associated lung adenocarcinoma transcript 1 (MALAT1) is a well-studied long noncoding RNA (lncRNA) known to promote tumorigenesis. MALAT1 overexpression in epithelial ovarian cancer cell lines results in tumor cell apoptosis inhibition, increased tumor cell inflammation and proliferation, as well as tumor cell invasion in the tumor microenvironment [4]. Thus, MALAT1 could serve as a potential detection molecular marker and therapeutic target in ovarian cancer. Bone morphogenetic proteins (BMPs) represent multi-functional growth factors involved in multiple signaling pathways. Fukuda, T. et al. [5] showed that the BMP signaling cascade promotes tumorigenesis in endometrial-derived cell lines. Furthermore, the in vitro use of the BMP inhibitors TWSG1 and LDN193189 trigger tumor-suppressing effects, emphasizing that BMP signaling merits further investigation.

The study of Ferreira, K.P. et al. [6] ought to determine both the gene and protein expression profiling of four membrane proteins, called tetraspanins (TSPANs), and their association with clinicopathological features in 117 vulvar squamous cell cancer patients. Apart from providing novel functional insights into these molecules in a rare gynecologic malignancy, the interesting results of this study underscore the potential of new therapeutic approaches for this ‘orphan disease’. In addition, a comprehensive literature review [7] summarized the available genomic data in vulvar squamous cell carcinoma, discussing the remarkable heterogeneity not only in the number and frequencies of reported mutations but also in the number of specific mutated genes.

Furthermore, this Special Issue included a review [8] with regards to cervical cancer pathogenesis and its potential diagnostic biomarkers and a systematic review [9] of 10 original studies with respect to the optimal patient-derived xenograft (PDX) models of cervical cancer. We would care to express our sincere gratitude to all the contributors to this Special Issue of Gynecologic Oncology for IJMC, providing their expert contributions, either as experimental or reviewed data, which will assuredly attract the scientific audience’s attention and undoubtedly encourage the publication of further, high-quality, research papers in the second edition of the Special Issue entitled: “Advances in Gynecological Cancers 2.0”.
